# Amino acid digestibility and nitrogen-corrected true metabolizable energy of mildly cooked human-grade vegan dog foods using the precision-fed cecectomized and conventional rooster assays

**DOI:** 10.1093/tas/txad020

**Published:** 2023-02-15

**Authors:** Leah J Roberts, Patricia M Oba, Pamela L Utterback, Carl M Parsons, Kelly S Swanson

**Affiliations:** Department of Animal Sciences, University of Illinois at Urbana-Champaign, Urbana, IL 61801; Department of Animal Sciences, University of Illinois at Urbana-Champaign, Urbana, IL 61801; Department of Animal Sciences, University of Illinois at Urbana-Champaign, Urbana, IL 61801; Department of Animal Sciences, University of Illinois at Urbana-Champaign, Urbana, IL 61801; Department of Animal Sciences, University of Illinois at Urbana-Champaign, Urbana, IL 61801; Division of Nutritional Sciences, University of Illinois at Urbana-Champaign, Urbana, IL 61801

**Keywords:** canine nutrition, nutrient digestion, pet food, rooster model

## Abstract

The pet food market is constantly changing and adapting to meet the needs and desires of pets and their owners. One trend that has been growing in popularity lately is the feeding of fresh, human-grade foods. Human-grade pet foods contain ingredients that have all been stored, handled, processed, and transported in a manner that complies with regulations set for human food production. While most human-grade pet foods are based on animal-derived ingredients, vegan options also exist. To our knowledge, no in vivo studies have been conducted to analyze the performance of human-grade vegan diets. Therefore, the objective of this study was to investigate the amino acid (AA) digestibility and nitrogen-corrected true metabolizable energy (TME_*n*_) of mildly cooked human-grade vegan dog foods using precision-fed cecectomized rooster and conventional rooster assays. Three commercial dog foods were tested. Two were mildly cooked human-grade vegan dog diets (Bramble Cowbell diet (BC); Bramble roost diet (BR)), while the third was a chicken-based extruded dog diet (chicken and brown rice recipe diet (CT)). Prior to the rooster assays, both mildly cooked diets were lyophilized, and then all three diets were ground. Diets were fed to cecectomized roosters to determine AA digestibility, while conventional roosters were used to determine TME_*n*_. All data were analyzed using the mixed models procedure of SAS (version 9.4). The majority of indispensable and dispensable AA across all diets had digestibilities higher than 80%, with a few exceptions (BC: histidine, lysine, threonine, and valine; BR: histidine). The only difference in indispensable AA digestibility among diets was observed with tryptophan, with its digestibility being higher (*P* = 0.0163) in CT than in BC. TME_*n*_ values were higher (*P* = 0.006) in BC and BR (4.55 and 4.66 kcal/g dry matter, respectively) than that in CT (3.99 kcal/g dry matter). The TME_*n*_/GE was also higher (*P* = 0.0193) in BR than in CT. Metabolizable energy (ME) estimates using Atwater factors accurately estimated the energy content of CT, but modified Atwater factors and the predictive equations for ME recommended by the National Research Council underestimated energy content. All calculations underestimated the measured TME_*n*_ values of BC and BR, with Atwater factors being the closest. Although testing in dogs is required, these data suggest that mildly cooked human-grade vegan dog diets are well-digested. Moreover, TME_*n*_ data suggest that existing methods and equations underestimate the ME of the mildly cooked human-grade vegan foods tested.

## INTRODUCTION

The bond between humans and their pets has continued to grow, leading to changes in pet care and feeding practices. These changes have affected the types of foods developed for and fed to pets. Some consumers have shifted away from extruded kibble diets containing traditional ingredients and instead have chosen to feed premium foods that contain novel proteins with a limited number of ingredients, or foods that are natural, organic, or human-grade (Euromonitor, 2022). Another diet trend gaining popularity is that of plant-based foods. Interest in vegetarian or vegan dog foods may be attributed to concerns about animal welfare, environmental sustainability, or perceptions of what a healthy diet contains (Deng and Swanson, 2015; Martens et al., 2019; Alexander et al., 2020; Knight et al., 2022).

Vegetarian and vegan foods may take any form, but must be carefully formulated and based on complementary ingredients such as grains, legumes, fruits, and vegetables to meet nutrient requirements. Formulating a nutritionally complete and balanced plant-based diet can be a challenge for pet food companies (Ingenpaß et al., 2021). A major concern of pet food producers is the quality of proteins present in vegan diets. Protein-rich ingredients are needed to provide indispensable amino acids (AA) and nitrogen that are necessary for maintaining proper physiological functions (Ingenpaß et al., 2021). Plant-based diets can take various forms, whether it be kibble, or mildly cooked food. Mildly cooked foods are appealing to consumers because they often resemble human food, making dog owners feel as though they are feeding their dogs just as well as themselves. Another option many pet owners are turning to are human-grade pet foods, which are products that contain ingredients that have been processed, handled, stored, and transported in a way that meets the standards set by 21 CFR part 117 and other federal human food laws specific to individual ingredients, facilities, and processing methods, therefore maintaining human-grade status (AAFCO, 2022). Human-grade pet foods must be clearly labeled with the intended species and use, with this information being more prominent than the term “human-grade” (AAFCO, 2022).

Very few studies have been performed to study vegan or vegetarian dog foods, but surveys have been conducted recently to investigate the potential health implications and palatability of vegan pet foods. One recent survey reported that dogs consuming vegan diets were generally healthier than dogs consuming conventional or raw meat diets (Knight et al., 2022). Another survey performed by Knight and Satchell (2021) reported that vegan dog and cat foods were equally as palatable as conventional or raw meat diets and that they do not compromise their welfare. Other researchers investigated the nutritional integrity and adequacy of vegan diets by measuring crude protein (CP) and AA concentrations in various dry and canned vegetarian dog and cat foods (Kanakubo et al., 2015). The results of that cross-sectional study revealed that six of the diets did not meet minimum AA recommendations. To our knowledge, however, no previous study has examined the in vivo AA digestibilities or metabolizable energy (ME) content of vegan or vegetarian dog foods.

Given the lack of testing on vegan pet diets, the primary objective of this experiment was to determine the AA digestibilities of commercial mildly cooked human-grade vegan dog foods using the precision-fed cecectomized rooster assay. Our secondary objective was to compare ME estimate calculations [National Research Council (NRC, 2006) equation; Atwater factors; modified Atwater factors] against nitrogen-corrected true metabolizable energy (TME_*n*_) values generated from the conventional rooster assay. Our hypothesis was that these vegan diets would have greater AA digestibilities than an extruded kibble diet and higher TME_*n*_ values than standard ME estimations due to their mild cooking conditions.

## MATERIALS AND METHODS

### Substrates

Two mildly cooked human-grade vegan diets [The Cowbell (BC) and The Roost (BR); Bramble Inc., New York, NY] and an extruded dry kibble diet [Life Protection Formula Chicken and Brown Rice Recipe (CT); Blue Buffalo, Wilton, CT] were tested in this study. The human-grade vegan diets tested were mildly cooked according to U.S. food and drug administration guidelines (21 CFR part 507), with standard operating procedures for ingredient weights and cooking temperatures and times (target temperature of 74°C for at least 5 min). All diets were formulated to meet all Association of American Feed Control Officials (AAFCO, 2021) nutrient profiles for adult dogs at maintenance (Table 1). Before analysis, Bramble diets were lyophilized (Dura-Dry MP microprocessor-controlled freeze-dryer; FTS Systems, Stone Ridge, NY), which was required to conduct the dosing for the rooster experiment. Following freeze-drying, all diets were ground through a 2-mm screen (Wiley mill Model 4; Thomas Scientific, Swedesboro, NJ).

**Table 1.** Chemical composition and metabolizable energy values of dog foods tested^*a*^

Item	CT^*b*^	BC^*c*^	BR^*d*^
Chemical composition			
DM^*e*^,%	92.46	95.34	95.70
	Dry matter basis		
Ash, %	7.64	5.53	5.53
CP, %	26.69	32.50	34.20
AHF, %	17.28	21.71	20.82
TDF, %	12.88	12.24	11.50
TIF, %	7.70	6.72	6.69
TSF, %	5.17	5.52	4.81
NFE^*f*^, %	35.51	28.02	27.95
GE, kcal/g	5.16	5.66	5.48
Metabolizable energy estimates			
ME (Atwater factors)^*g*^, kcal/g	4.04	4.37	4.36
ME (modified Atwater factors)^*h*^, kcal/g	3.65	3.96	3.94
ME (NRC equation)^*i*^, kcal/g	4.05	4.37	4.38

^*a*^ *n* = 4 roosters per treatment.

*b*CT = Life Protection Formula Chicken and Brown Rice, Blue Buffalo, Wilton, CT. Ingredients: deboned chicken, chicken meal, brown rice, barley, oatmeal, pea starch, flaxseed, chicken fat, dried tomato pomace, natural flavor, peas, pea protein, salt, potassium chloride, dehydrated alfalfa meal, potatoes, dried chicory root, pea fiber, alfalfa nutrient concentrate, calcium carbonate, choline chloride, DL-methionine, preserved with mixed tocopherols, dicalcium phosphate, sweet potatoes, carrots, garlic, zinc amino acid chelate, zinc sulfate, vegetable juice for color, ferrous sulfate, vitamin E supplement, iron amino acid chelate, blueberries, cranberries, barley grass, parsley, turmeric, dried kelp, yucca schidigera extract, niacin (vitamin B_3_), glucosamine hydrochloride, calcium pantothenate (vitamin B_5_), copper sulfate, biotin (vitamin B_7_), L-ascorbyl-2-polyphosphate, L-lysine, L-carnitine, vitamin A supplement, copper amino acid chelate, manganese sulfate, taurine, manganese amino acid chelate, thiamine mononitrate (vitamin B_1_), riboflavin (vitamin B_2_), vitamin D_3_ supplement, vitamin B_12_ supplement, pyridoxine hydrochloride (vitamin B_6_), calcium iodate, dried yeast, dried *Enterococcus faecium* fermentation extract, dried *Trichoderma longibrachiatum* fermentation extract, dried *Bacillus subtilis* fermentation extract, folic acid (vitamin B_9_), sodium selenite, oil of rosemary.

^*c*^BC = The Cowbell, Bramble Inc., New York, NY. Ingredients: organic pea protein, lentil, sweet potato, carrots, organic sunflower oil, organic flax oil, peas, apples, malt extract, potato starch, nutrient mix [choline chloride, potassium chloride, L-methionine, tricalcium phosphate, taurine], vitamins [D-calcium pantothenate, riboflavin, niacin, vitamin B_12_, vitamin A acetate, vitamin E supplement, folic acid, thiamine mononitrate, pyridoxine hydrochloride, vitamin D_2_ supplement], trace minerals [zinc proteinate, iron proteinate, copper proteinate, manganese proteinate, calcium iodate, selenium yeast], nutritional yeast, caramel color, tricalcium phosphate, potassium chloride, sodium phosphate, magnesium, salt.

^*d*^BR = The Roost, Bramble Inc., New York, NY. Ingredients: organic pea protein, long grain brown rice, potato, garbanzo beans, carrots, organic sunflower oil, peas, butternut squash, blueberries, malt extract, potato starch, nutrient mix [choline chloride, potassium chloride, L-methionine, tricalcium phosphate, taurine], vitamins [D-calcium pantothenate, riboflavin, niacin, vitamin B_12_, vitamin A acetate, vitamin E supplement, folic acid, thiamine mononitrate, pyridoxine hydrochloride, vitamin D_2_ supplement], trace minerals [zinc proteinate, iron proteinate, copper proteinate, manganese proteinate, calcium iodate, selenium yeast], nutritional yeast, tricalcium phosphate, potassium chloride, sodium phosphate, magnesium, salt.

^*e*^DM = dry matter, CP = crude protein, AHF = acid-hydrolyzed fat, TDF = total dietary fiber, TIF = total insoluble fiber, TSF = total soluble fiber, NFE = nitrogen-free extract, GE = gross energy.

^*f*^NFE = 100% − (% CP in DM + % AHF in DM + % TDF in DM + % ash in DM).

^*g*^ME (Atwater factors; kcal/g) = [(4 × CP in DM) + (9 × AHF in DM) + (4 × NFE in DM)]/100.

^*h*^ME (modified Atwater factors; kcal/g) = [(3.5 × CP in DM) + (8.5 × AHF in DM) + (3.5 × NFE in DM)]/100.

^*i*^ME (NRC, 2006 equation):.

-Gross energy (GE) (kcal) = (5.7 × % CP in DM) + (9.4 × % AHF in DM) + [4.1 × (% NFE + % TDF in DM)]

-Energy digestibility (ED) (%): 96.6 − (0.95 × % TDF in DM)

-Digestible energy (DE): (kcal GE × ED)/100

-ME (NRC) (kcal/g) = [kcal DE − (1.04 × % CP in DM)]/100

### Cecectomized and Conventional Rooster Assays

The protocol for the cecectomized and conventional rooster assays, including all animal handling, housing, and surgical procedures, was reviewed and approved by the Institutional Animal Care and Use Committee at the University of Illinois at Urbana-Champaign prior to experimentation. First, a precision-fed rooster assay utilizing Single Comb White Leghorn roosters (1.5 to 2.5 yr old, 2.5 to 3 kg BW) was conducted as described by Parsons (1985) to determine the AA digestibility of the test diets. Prior to the study, a cecectomy was performed on roosters under general anesthesia according to the procedures of Parsons (1985). All roosters were given at least 8 wk to recover from surgery before being used in experiments. In that study, 12 cecectomized roosters were randomly assigned to the three test diets (*n* = 4 roosters/diet). In the second assay, 12 conventional roosters were randomly assigned to the three test diets (*n* = 4 roosters/diet) for TME_*n*_ calculations.

In both assays, after 26 h of feed withdrawal and ad libitum water, roosters were tube-fed 15 g of test diets + 15 g of corn. Following crop intubation, excreta was collected for 48 h on plastic trays placed under each individual cage. Excreta samples then were lyophilized, weighed, and ground through a 0.25-mm screen prior to analysis. Endogenous corrections for AA were made using five additional cecectomized roosters that had been fasted for 48 h. AA digestibilities were calculated using the method described by Engster et al. (1985). All birds were housed individually in cages (27.9 cm wide × 50.8 cm long × 53.3 cm high) with raised wire floors. They were kept in an environmentally controlled room (approximately 23.9°C, 17 h light:7 h dark). Before the start of the experiment, feed and water were supplied for ad libitum consumption.

### Chemical Analyses

The diets and rooster excreta were analyzed for dry matter (DM; 105°C) and ash according to AOAC (2006) with the organic matter being calculated. Nitrogen was measured and CP was calculated using a Leco Nitrogen/Protein Determinator (Model FP-2000, Leco Corporation, St. Joseph, MI) according to AOAC (2006; method 982.30E). The total dietary fiber (TDF) of each diet was determined according to Prosky et al. (1985). Acid-hydrolyzed fat (AHF) concentrations of the diets were determined by acid hydrolysis according to AACC (1983), followed by diethyl ether extraction (Budde, 1952). The AA were measured at the University of Missouri Experiment Station Chemical Laboratories (Columbia, MO) according to AOAC (2006; method 982.30E). The gross energy (GE) of the diets and rooster excreta was measured using a bomb calorimeter (Model 6200; Parr Instrument Com., Moline, IL).

### Amino Acid Digestibility Calculations

Basal endogenous AA concentrations were determined using roosters that were fasted for 48 h and then standardized AA digestibility values were calculated by the method of Engster et al. (1985) using the equations below.


$$\begin{array}{l} \text{AA digestibility of diet }(\text{\%})=\left[\frac{\begin{array}{l} \text{AA consumed}-\text{AA excreted by fed birds}\\ \;+\text{AA excreted by fasted birds} \end{array}}{\text{AA consumed}}\right]\times 100 \end{array}$$

where AA consumed (g) = diet intake (g) × AA in diet (%); AA excreted by fed birds (g) = excreta output (g) × AA in excreta (%); AA excreted by fasted birds = excreta output (g) × AA in excreta (%). The AA digestibility values for test diets were then calculated by difference using the equation:


$$\begin{array}{l} \text{AA digestibility of test diet }\left(\%\right)\\ =\text{AA digestibility of ground corn reference diet}\\ -\left[\frac{\begin{array}{l} (\text{standardized AA digestibility}\\ \text{of ground corn reference diet}\\ -\text{standardized AA digestibility of}\\ \text{test diet mixture with corn}) \end{array}}{\begin{array}{l} \text{proportion of test diet AA substituted}\\ \text{into the test diet mixture with corn} \end{array}}\right]. \end{array}$$

### *Nitrogen-Corrected True Metabolizable Energy (TME*_*n*_ *) Calculations*

The calculation of TME_*n*_ was performed according to Parsons et al. (1982). The TME_*n*_ values, corrected for endogenous energy excretion using many fasted birds over many years, were calculated using the following equation:


$$\begin{array}{l} {\text{TME}}_{n}\text{ of diet (kcal/g)}\\ \text{=}\left[\frac{\begin{array}{l} \text{GE consumed}(\text{GE excreted by fed birds}\\ +8.22\;\times \text{nitrogen retained by fed birds})\\ +(\text{GE excreted by fasted birds}\\ +8.22\;\times \text{nitrogen retained by fasted birds}) \end{array}}{\text{feed intake }(\text{g})}\right] \end{array}$$

where GE consumed (kcal) = diet intake (g) × GE of diet (kcal/g); GE excreted by fed or fasted birds (kcal) = excreta output (g) × GE of excreta (kcal/g); 8.22 = GE (kcal) of uric acid per g of nitrogen (Hill and Anderson, 1958); nitrogen retained by fed or fasted birds (g) = diet intake (g) × diet nitrogen (%) − excreta output (g) × excreta nitrogen (%). The TME_*n*_ values were then calculated by difference as shown below.


$$\begin{array}{l} {\text{TME}}_{n}(\text{kcal}/\text{g})\\ ={\text{TME}}_{n}\text{ of ground corn reference diet}\\ -\left[\frac{\begin{array}{l} {\text{TME}}_{n}\text{ of ground corn reference diet}\\ {\text{TME}}_{n}\text{ of test diet} \end{array}}{\begin{array}{l} \text{proportion of test diet}\\ \text{substituted into the corn reference diet} \end{array}}\right] \end{array}$$

The AA digestibility data derived from the current study were used to estimate the digestible indispensable AA concentrations of the diets tested on a weight basis (g digestible AA/100 g diet). The AA digestibility data and TME_*n*_ data derived from the current study were used to estimate the digestible indispensable AA concentrations of the diets tested on a caloric basis (g digestible AA/1,000 kcal ME).

### Metabolizable Energy Calculations

To compare against TME_*n*_ data, ME estimates were performed according to NRC (2006) calculations [using TDF instead of crude fiber (CF) values], Atwater factors (Atwater, 1902), and modified Atwater factors. Nitrogen-free extract (NFE) values (using TDF instead of CF values) and ME were calculated using the following equations:

(a) NFE (%) = 100% − (% CP in DM + % AHF in DM + % TDF in DM + % Ash in DM)
(b) ME (Atwater factors; kcal/g) = [(4 × CP in DM) + (9 × AHF in DM) + (4 × NFE in DM)]/100
(c) ME (modified Atwater factors; kcal/g) = [(3.5 × CP in DM) + (8.5 × AHF in DM) + (3.5 × NFE in DM)]/100
(d) ME (NRC, 2006 equation; kcal/g) =

1. Gross energy (kcal) = (5.7 × % CP in DM) + (9.4 × % AHF in DM) + [4.1 × (% NFE + % TDF in DM)]
2. Energy digestibility (%): 96.6 – (0.95 × % TDF in DM)
3. Digestible energy (DE): (kcal GE × energy digestibility)/100
4. ME (NRC) (kcal/g) = [kcal DE – (1.04 × % CP in DM)]/100

Because the CF assay does not accurately measure dietary fiber, it should not be used for estimating the fiber content of pet foods (Fahey et al., 2019). Therefore, the TDF assay that allows the measurement of both soluble and insoluble fiber fractions and is a much better fiber estimate, was used in the equations above. Using TDF to estimate NFE has been shown to have a high correlation with starch content in dog foods (de-Oliveira et al., 2012).

### Statistical Analyses

All data were analyzed using the Mixed Models procedure of SAS (version 9.4; SAS Institute, Cary, NC). Diets were considered to be a fixed effect, and roosters were considered to be a random effect. Tukey’s multiple comparison analysis was used to compare the least square means for experiment-wise error. Differences were considered significant when *P* < 0.05.

## RESULTS

### Chemical Composition

The analyzed nutrient composition of all diets is presented in Table 1, with the DM values for BC and BR representing the diets after the freeze-drying process. On a DM basis, ash content was slightly higher in the CT diet (7.64%) than in BC or BR diets (5.53%). The BR and BC diets had higher CP concentrations (34.20% and 32.50%, respectively) than the CT diet (26.69%). The AHF and GE were slightly higher in the BR and BC diets than in the CT diet (20.82% and 21.71% vs. 17.28%; 5.48 kcal/g and 5.66 kcal/g vs. 5.16 kcal/g). TDF concentration was higher in the CT diet (12.88%) than in the BC (12.24%) and BR (11.50%) diets. The CT diet contained the highest TIF (7.70%) and NFE (35.51%) concentrations. The BC diet contained the highest TSF concentration (5.52%). Dietary ME estimates based on modified Atwater factors, Atwater factors, and the NRC equation (NRC, 2006) were higher in the BC and BR diets than those estimated for the CT diet.

Dietary concentrations of indispensable and dispensable AA and inclusion levels recommended by AAFCO for adult dogs at maintenance (AAFCO, 2022) are presented in Table 2. These data reveal that all three diets contained concentrations of indispensable AA above the AAFCO recommended inclusion levels based on a weight (% AA, DM basis) and caloric (g AA/1,000 kcal ME) basis. Because sulfur-containing AA are typically the limiting AA of legumes and pulses, and because some have attempted to link these ingredients with dilated cardiomyopathy in dogs, it is important to note that the vegan diets (BC; BR) tested easily exceeded the AAFCO methionine and methionine-cystine recommendations and contained higher taurine concentrations than the CT diet.

**Table 2.** Indispensable and dispensable amino acid (AA) concentrations (% dry matter and g/1,000 kcal) of dog foods tested

	% dry matter				g/1,000 kcal			
Item	AAFCO^*a*^	CT^*b*^	BC	BR	AAFCO	CT	BC	BR
Indispensable AA								
Arginine	0.51	1.78	2.59	2.83	1.28	4.88	6.54	7.18
Histidine	0.19	0.61	0.78	0.84	0.48	1.67	1.97	2.13
Isoleucine	0.38	0.98	1.48	1.62	0.95	2.68	3.74	4.11
Leucine	0.68	1.78	2.57	2.78	1.7	4.88	6.49	7.06
Lysine	0.63	1.65	2.30	2.42	1.58	4.52	5.81	6.14
Methionine	0.33	0.74	0.63	0.65	0.83	2.03	1.59	1.65
Methionine-cystine	0.65	1.11	0.96	1.04	1.63	3.07	2.45	2.66
Phenylalanine	0.45	1.06	1.71	1.86	1.13	2.90	4.32	4.72
Phenylalanine-tyrosine	0.74	1.91	2.81	3.03	1.85	5.23	7.10	7.69
Threonine	0.48	0.96	1.21	1.27	1.2	2.63	3.06	3.22
Tryptophan	0.16	0.23	0.27	0.33	0.4	0.63	0.68	0.84
Valine	0.49	1.18	1.63	1.76	1.23	3.23	4.12	4.47
Selected dispensable AA								
Alanine	–	1.58	1.42	1.50	–	4.33	3.59	3.81
Aspartic acid	–	2.22	3.77	3.97	–	6.08	9.52	10.08
Cysteine	–	0.38	0.34	0.40	–	1.04	0.86	1.02
Glutamic acid	–	4.22	5.38	5.91	–	11.56	13.59	15.00
Glycine	–	2.11	1.33	1.39	–	5.78	3.36	3.53
Proline	–	1.56	1.35	1.48	–	4.27	3.41	3.76
Serine	–	0.98	1.51	1.59	–	2.68	3.81	4.04
Tyrosine	–	0.85	1.10	1.17	–	2.33	2.78	2.97
Taurine	–	0.27	0.42	0.38	–	0.74	1.06	0.96

^*a*^Association of American Feed Control Officials (AAFCO, 2022) for adult dogs at maintenance.

^*b*^CT = Life Protection Formula Chicken and Brown Rice (Blue Buffalo, Wilton, CT), BC = The Cowbell (Bramble Inc., New York, NY), BR = The Roost (Bramble Inc., New York, NY).

### Rooster Assays

The majority of the indispensable AA had digestibility values greater than 80% in all three diets (Table 3). The exceptions were histidine (65.66%), lysine (75.28%), threonine (72.52%), and valine (77.03%) for the BC diet and histidine (76.49%) for the BR diet. The tryptophan digestibility of the CT diet (100.03%) was higher (*P* < 0.05) than that of the BC diet (92.76%), but all other indispensable AA were not different (*P* > 0.05) among diets. Many of the dispensable AA also had digestibility values greater than 80%, with the exception of aspartic acid (77.37%) and cysteine (69.76%) in the CT diet, alanine (74.74%), aspartic acid (79.19%), cysteine (42.35%), proline (72.82%), and serine (78.33%) in the BC diet, and cysteine (60.50%) in the BR diet. Digestibility of aspartic acid was higher (*P* < 0.05) in the BR diet (85.84%) than the CT diet (77.37%), but all other dispensable AA were not different (*P* > 0.05) among diets.

**Table 3.** Amino acid (AA) digestibilities (%) of dog foods using the precision-fed cecectomized rooster assay^*a*^

Item	CT^*b*^	BC	BR	SEM	*P*-value
Indispensable AA					
Arginine	91.28	89.62	92.05	1.75	0.6189
Histidine	81.48	65.66	76.49	4.86	0.1159
Isoleucine	87.99	81.98	87.56	2.22	0.1577
Leucine	87.99	81.38	86.97	2.32	0.1502
Lysine	83.35	75.28	82.71	2.61	0.1034
Methionine	89.15	84.90	87.50	1.72	0.2659
Phenylalanine	87.59	83.99	88.81	2.13	0.2992
Threonine	81.15	72.52	81.60	4.03	0.2511
Tryptophan	100.03^*a*^	92.86^*b*^	96.86^*ab*^	1.38	0.0163
Valine	83.80	77.03	83.97	3.01	0.2311
Selected dispensable AA					
Alanine	85.09	74.74	82.00	2.92	0.0837
Aspartic acid	77.37^*b*^	79.19^*ab*^	85.84^*a*^	1.84	0.0236
Cysteine	69.76	42.35	60.50	8.34	0.1135
Glutamic acid	86.02	84.47	89.31	1.67	0.1688
Glycine	94.94	83.15	93.89	5.59	0.3041
Proline	83.84	72.85	82.48	4.20	0.1870
Serine	80.47	78.33	84.16	3.50	0.5182
Tyrosine	85.90	81.41	86.48	2.49	0.3341
TME_*n*_^*c*^					
TME_*n*_, kcal/g	3.99^*b*^	4.55^*a*^	4.64^*a*^	0.0818	0.0006
TME_*n*_/GE, %	77.3^*b*^	80.4^*ab*^	84.7^*a*^	1.477	0.0193

^*a*^ *n* = 4 roosters per treatment.

^*b*^CT = Life Protection Formula Chicken and Brown Rice (Blue Buffalo, Wilton, CT), BC = The Cowbell (Bramble Inc., New York, NY), BR = The Roost (Bramble Inc., New York, NY).

^*c*^TME_*n*_ = nitrogen-corrected true metabolizable energy.

^*ab*^Within a row, means lacking a common superscript differ (*P* < 0.05).

TME_*n*_ values were higher (*P* < 0.05) in the BC and BR diets (4.55 and 4.66 kcal/g, respectively) than in the CT diet (3.99 kcal/g) (Table 3). TME_*n*_ expressed as a percentage of GE was higher (*P* < 0.05) in the BR diet (84.7%) than in the CT diet (77.3%). The TME_*n*_/GE of the BC diet was not different than that of the BR or CT diets (*P* > 0.05). The modified Atwater factors and NRC equations underestimated ME for all three diets. The Atwater factors underestimated ME for BR and BC, but were a close estimate of CT.

Based on the AA digestibility data and TME_*n*_ data derived from the current study, estimates of digestible AA on a weight (g digestible AA/100 g diet) and calorie content (g digestible AA/1,000 kcal ME) basis for all diets are presented in Table 4. On a weight basis, the BC and BR diets had higher digestible AA estimates than the CT diet for all AA, except for methionine and methionine-cystine. Similar results were noted when caloric content was considered, but the differences were not as great.

**Table 4.** Digestible indispensable amino acid concentrations of dog foods tested^*a*^

	g/100 g of diet			g/1,000 kcal of diet		
Item	CT^*b*^	BC	BR	CT	BC	BR
Arginine	1.51	2.21	2.49	4.08	5.10	5.62
Histidine	0.46	0.49	0.61	1.24	1.12	1.38
Isoleucine	0.80	1.16	1.36	2.17	2.66	3.06
Leucine	1.45	1.99	2.31	3.93	4.59	5.21
Lysine	1.28	1.65	1.92	3.46	3.80	4.32
Methionine	0.61	0.51	0.54	1.64	1.17	1.22
Methionine-cystine	0.85	0.64	0.77	2.30	1.48	1.74
Phenylalanine	0.86	1.37	1.58	2.33	3.15	3.56
Phenylalanine-tyrosine	1.54	2.22	2.55	4.17	5.12	5.74
Threonine	0.72	0.83	1.00	1.96	1.92	2.24
Tryptophan	0.16	0.24	0.31	0.43	0.56	0.70
Valine	0.41	1.19	1.41	1.11	2.75	3.18

^*a*^Values are based on the measured AA digestibilities and TME_*n*_ of the dog foods tested.

^*b*^CT = Life Protection Formula Chicken and Brown Rice (Blue Buffalo, Wilton, CT), BC = The Cowbell (Bramble Inc., New York, NY), BR = The Roost (Bramble Inc., New York, NY).

## DISCUSSION

In 2020, the vegan pet food market was worth $8.7 billion, and in the next 6 years, it is projected to reach $15.7 billion, which is a growth rate of 7.7% (Knight et al., 2022). As the market for plant-based dog foods continues to grow, so does the need for research into this diet format. The amount and quality of protein are extremely important to consider when formulating a complete and balanced diet. The vegan diets tested in the current study easily exceeded the AAFCO (AAFCO, 2022) and European Pet Food Industry Nutritional Guidelines (FEDIAF, 2021) recommendations for CP and all indispensable AA, but that is not always the case with this diet category. A recent study evaluated extruded vegan dog and cat diets available in Brazil (Zafalon et al., 2020). The three vegan dog foods contained 24.36%, 27.96%, and 30.04% CP, which exceeds the guidelines provided by AAFCO (18% CP for adult dogs; 22.5% for growing and reproducing dogs) and The European Pet Food Industry Nutritional Guidelines (FEDIAF, 2021) for adult dogs (21% CP). One of the diets, however, contained slightly lower CP concentrations than that recommended by FEDIAF for the young growing (<14 wk old) and reproducing dogs (25% CP). In that study, all dietary AA concentrations exceeded recommendations set by AAFCO and FEDIAF, except for methionine in one of the foods (0.41%), which was lower than that recommended by FEDIAF (0.46% for adults).

Dietary CP concentrations, protein quality (i.e., AA profile), and nutrient digestibilities are key considerations and potential concerns of vegan diets. Although pet owner surveys about the reasons for feeding of vegan diets and chemical analysis of commercial vegan diets have been done in the past, nobody has evaluated their in vivo digestibility or ME content. Therefore, in addition to chemically analyzing the test diets in the current study, our initial analyses involved the use of the cecectomized rooster assay to determine AA digestibilities and conventional roosters to estimate energy content. Accurately measuring the nutrient digestibility and energy content of diets not only provides useful information about the specific nutrients in question but also demonstrates their relationship with caloric density, which is important for diet formulation and feeding guidelines. The cecectomized rooster assay is a well-accepted model for measuring the AA digestibility of individual ingredients or complete and balanced pet foods because it minimizes the effects of microbial fermentation. Data from this model have been shown to have a high correlation to data generated by the ileal-cannulated dog model (Johnson et al., 1998). In the past, ileal-cannulated dogs were commonly used for measuring the macronutrient and AA digestibilities of dog foods (Bednar et al., 2000; Burkhalter et al., 2001; Faber et al., 2010). However, in recent years, the cecectomized rooster assay has become a more popular alternative because it can be used to evaluate individual ingredients, experimentation requires a shorter length of time, and it comes with fewer animal welfare concerns and expense than ileal-cannulated dogs (Deng and Swanson, 2015). Conventional roosters have been used in past studies to estimate the TME_*n*_ of individual ingredients and complete and balanced pet foods (Knapp et al., 2010; de Godoy et al., 2014; Oba et al., 2020).

Most vegan diets rely on pulses, including beans, lentils, and peas, to provide the majority of CP and indispensable AA. The BC diet tested in the current study relies on organic pea protein, lentils, and whole peas for protein, while the BR diet tested contains organic pea protein, garbanzo beans, and whole peas as primary protein sources. A previous study used the cecectomized rooster model to evaluate the AA digestibilities of pulse ingredients, including black bean grits, garbanzo beans, green lentils, navy bean powder, and yellow peas (Reilly et al., 2020). In general, the indispensable AA were highly digestible, with digestibility values ranging from 80% to 90% for all pulse ingredients. The exception was methionine, which had a digestibility ranging from 72.9% (green lentils) to 81.9% (garbanzo beans). The same response was not noted in the current study, as methionine digestibilities were about 85% to 87.5% in the vegan diets, and not statistically different than that of the control diet. In addition to AA digestibility, the Reilly et al. (2020) study estimated digestible indispensable amino acid scores (DIAAS)-like values using dog and cat references to determine the protein quality of each pulse tested. For the five pulse ingredients evaluated, methionine and tryptophan were determined to be the first-limiting AA depending on the life stage and species in question. The authors concluded that pulse ingredients are viable protein sources in pet foods, but the use of complementary protein sources is recommended to avoid AA imbalances. That study was different than the current study in that it evaluated the AA digestibilities of individual pulses and legumes rather than the complete and balanced diets tested herein. Together, these studies describe how well these ingredients, and diets largely composed of them, are digested.

Other studies have used ileal-cannulated dogs or cecectomized roosters to evaluate the AA digestibilities of complete and balanced dog diets containing pulses and legumes as primary protein sources, which have relevance to the current study. In one ileal-cannulated dog study, ileal and total tract nutrient digestibility of dry, extruded diets containing different forms of soy protein (soybean meal, soy flour, and soy protein concentrate) were compared with a dry, extruded diet based on poultry meal (Clapper et al., 2001). In that study, the apparent ileal digestibility of the most indispensable AA was higher (*P* < 0.05) in diets containing soy proteins than in poultry meals, with soy protein concentrates performing at the highest level. The studies published by Clapper et al. (2001) and Reilly et al. (2020) demonstrate that pulse- and legume-based ingredients and diets may perform well when processed appropriately and combined with complementary proteins. Because animal-based proteins tend to vary due to the carcass components included, processing methods, and other factors (Murray et al., 1997; Clapper et al., 2001), plant-based proteins may provide benefits in regard to diet consistency. In the current study, the test diets were mildly cooked and human-grade. Therefore, the processing and ingredient sourcing was very different than that of the dry, extruded diets utilized in the studies by Reilly et al. (2020) and Clapper et al. (2001).

The type and level of heat processing is also an important consideration because over-processing can damage AA and reduce nutrient digestibility. Because the human-grade vegan diets tested in the current study are mildly cooked, high digestibilities were anticipated. Cecectomized roosters have been used to test processing effects on individual ingredients and complete and balanced diets undergoing mild processing. In one study, Oba et al. (2019) investigated nutrient digestibility, AA digestibility, and TME_*n*_ of chicken-based ingredients that had undergone various levels of processing (i.e., chicken meal, raw chicken, retorted chicken, and steamed chicken) using the precision-fed cecectomized rooster assay. The results showed that steamed chicken, which had undergone the least amount of processing, had the highest indispensable AA digestibilities, with most being over 90% digestible. TME_*n*_ and DIAAS-like values were also significantly higher in the less-processed chicken-based ingredients compared with the chicken meal. These results support the idea that utilizing mild cooking rather than heavy processing will yield higher AA digestibility. The vegan diets tested in the current study were only cooked for approximately 5 min at a temperature of 74°C, which is mild for pet foods. Those conditions were similar to the steaming process utilized in the study conducted by Oba et al. (2019) and that was shown to provide the highest AA digestibility (vs. chicken meal, retorted chicken, and raw chicken).

In another study, Oba et al. (2020) used cecectomized and conventional roosters to measure AA digestibilities and TME_*n*_ of animal-based human-grade dog foods, respectively. In that study, six human-grade diets based on beef and potato, chicken and white rice, fish and sweet potato, lamb and brown rice, turkey and whole wheat macaroni, and venison and squash were evaluated. All of the diets tested were shown to have high AA digestibility, with all indispensable AA being greater than 85% digestible and some being over 90% digestible (Oba et al., 2020). Because the ingredient composition, nutrient composition, processing conditions, and other factors influence the digestibility of a diet, different diet formats and strategies should undergo testing as well. In the present study, two of the diets tested were fresh, mildly cooked, human-grade vegan diets, while the third diet was a meat-based extruded kibble, which acted as a high-quality kibble control. High AA digestibilities and TME_*n*_ values were observed in the vegan diets, which is consistent with the previous study on human-grade dog foods (Oba et al., 2020).

The TME_*n*_ data generated in the current study revealed that both vegan diets tested (BC and BR) had higher energy content than the control diet, and that BR had a higher TME_*n*_ expressed as a percentage of GE than CT. Atwater factors, modified Atwater factors, and the NRC predictive equation were also used to estimate ME and were shown to underestimate ME for the two human-grade vegan diets. The TME_*n*_ values measured for vegan diets (BC and BR) were approximately 15% greater than the estimates resulting from the modified Atwater factors that AAFCO (AAFCO, 2022) recommends and approximately 5% greater than the estimates resulting from the NRC equation (NRC, 2006) and the Atwater factors that are used for human foods. In comparison, the Atwater factors and estimates from the NRC equation (NRC, 2006) were quite similar to the TME_*n*_ values of the CT diet, with the modified Atwater factors underestimating the energy content of the CT diet by about 9%. As has been reported previously (Oba et al., 2020), the current equations used to estimate ME do not provide accurate estimates for highly digestible diets, especially mildly cooked human-grade foods.

Atwater factors (4, 9, and 4 kcal/g for proteins, fats, and digestible carbohydrates, respectively) are based on the heat of combustion and then corrected for losses through digestion, absorption, and urinary excretion (Atwater, 1902). These factors were created to estimate the ME of foods consumed by humans and were calculated based on the following digestibility coefficients: 96% for proteins and fats and 91% for carbohydrates. In the past, those digestibility values were higher than that present in most traditional commercial pet foods, resulting in an overestimation of ME. Because of this, the NRC (1985) suggested that digestibility coefficients of 80%, 90%, and 85% for protein, fat, and digestible carbohydrate, respectively, were more appropriate for commercial dog foods. That led to the use of modified Atwater factors (3.5, 8.5, and 3.5 kcal/g for proteins, fats, and digestible carbohydrates, respectively) that were accepted and are still used by AAFCO, FEDIAF, and regulatory agencies for determining ME of dog and cat foods. Even though modified Atwater factors may provide an accurate ME estimate of many pet foods, their usefulness is limited when applied to premium or super-premium diets that have low amounts of dietary fiber or connective tissue and have a high digestibility (Laflamme, 2001; Asaro et al., 2017; Castrillo et al., 2009; Oba et al., 2020). Even though it has no regulatory authority, NRC (2006) recommends using Atwater factors for estimating the ME of homemade dog and cat foods and commercial products that are highly digestible.

Based on the TME_*n*_ data generated for mildly cooked, human-grade diets in the current study and a recent study (Oba et al., 2020), the factors and equations used to estimate ME of these foods should be re-evaluated by regulatory agencies, companion animal nutritionists, and veterinarians. These estimates not only impact feeding guidelines and the food intake of pets, but the abundance of essential nutrients on a caloric basis (unit/1,000 kcal ME). This issue is further complicated by poor estimates of nutrient digestibility. Because the current study measured indispensable AA digestibility and TME_*n*_, estimates for digestible AA on a weight basis (g digestible AA/100 g diet) and caloric basis (g digestible AA/1,000 kcal ME) were possible. These values are much more useful and practical than simply measuring the nutrients and GE of diets.

Although diets containing pulses and legumes have been on the market for decades and shown to have high AA digestibility, such diets have been accused of contributing to low taurine status and the development of dilated cardiomyopathy in dogs. Over the past few years, many research groups have conducted prospective randomized studies to test if or how legume-based and/or grain-free diets impact the taurine status or biomarkers of dilated cardiomyopathy in dogs, but without significant findings (Donadelli et al., 2020; McCauley et al., 2020; Pezzali et al., 2020; Reilly et al., 2021). Moreover, no significant association between grain-free diet sales and dilated cardiomyopathy incidence rates was identified when data over the past two decades were examined (Quest et al., 2022). Despite the lack of evidence linking legumes and pulses with dilated cardiomyopathy in the scientific literature, the media and federal regulators have heightened the attention that these ingredients receive, with many veterinarians and consumers being fearful of their presence in dog foods. The data from the current study demonstrate that the vegan diets tested contained sufficient sulfur-containing AA, contained more taurine than the animal-based control diet, and that indispensable AA digestibilities were high and similar to that of the animal-based control diet. All indispensable AA were present at concentrations above those recommended by AAFCO (AAFCO, 2022) both on a weight (% AA, DM basis) and caloric basis (g AA/1,000 kcal ME). Lastly, the AA digestibility data and TME_*n*_ data also allowed for the estimates of digestible AA on a weight (g digestible AA/100 g diet) and caloric (g digestible AA/1,000 kcal ME) basis.

To conclude, the mildly cooked human-grade vegan diets tested in the current study performed well, easily meeting the CP and AA needs of dogs according to AAFCO and FEDIAF, having high AA digestibilities, and having high energy contents that exceeded current ME equations and estimates. With a few exceptions, all indispensable AA had digestibilities exceeding 80% for all diets tested. Even though tryptophan digestibility was higher in CT than BC, its digestibility was >92% in all diets tested. The inability of all energy predictive equations to accurately estimate ME of the mildly cooked, human-grade diets, which agrees with a previous study testing similar diets, suggests that these equations should be re-evaluated by regulatory agencies, companion animal nutritionists, and veterinarians. Even though the mildly cooked, human-grade vegan dog foods performed very well in this study using the cecectomized and conventional rooster assays, testing their acceptability, nutrient digestibility, and stool quality performance in dogs is suggested.
